# Spatial Attention and the Effects of Frontoparietal Alpha Band Stimulation

**DOI:** 10.3389/fnhum.2016.00658

**Published:** 2017-01-24

**Authors:** Martine R. van Schouwenburg, Theodore P. Zanto, Adam Gazzaley

**Affiliations:** ^1^Departments of Neurology, Physiology and Psychiatry, University of California, San FranciscoSan Francisco, CA, USA; ^2^Neuroscape, University of California, San FranciscoSan Francisco, CA, USA; ^3^Department of Psychology, University of AmsterdamAmsterdam, Netherlands

**Keywords:** alpha oscillations, coherence, connectivity, transcranial alternating current stimulation, visual attention

## Abstract

A frontoparietal network has long been implicated in top-down control of attention. Recent studies have suggested that this network might communicate through coherence in the alpha band. Here we aimed to test the effect of coherent alpha (8–12 Hz) stimulation on the frontoparietal network. To this end, we recorded behavioral performance and electroencephalography (EEG) data while participants were engaged in a spatial attention task. Furthermore, participants received transcranial alternating current stimulation (tACS) over the right frontal and parietal cortex, which oscillated coherently in-phase within the alpha band. Compared to a group of participants that received sham stimulation, we found that coherent frontoparietal alpha band stimulation altered a behavioral spatial attention bias. Neurally, the groups showed hemispheric-specific differences in alpha coherence between the frontal and parietal-occipital cortex. These results provide preliminary evidence that alpha coherence in the frontoparietal network might play a role in top-down control of spatial attention.

## Introduction

To navigate through our complex, high-interference environment, one needs to prioritize relevant information and suppress irrelevant information. Spatial attention tasks are widely used to study this phenomenon in the laboratory. In these tasks, a cue often informs the participant *where* relevant information is going to appear and many studies have shown that people are faster to respond to a target stimulus when they are cued towards its location, compared to when the target stimulus appears at an uncued location (Posner, 1980; Posner and Petersen, 1990). Although much research has identified frontal and parietal regions involved in such visuospatial orienting (Corbetta and Shulman, 2002; Noudoost et al., 2010), it remains unclear how these regions bias attention to spatial locations.

Neural recordings have established an important role for alpha (8–12 Hz) oscillations in spatial attention. In visual cortex, alpha activity decreases contralateral to the attended location, while it increases ipsilateral to the attended location (Worden et al., 2000; Sauseng et al., 2005). Such findings have led to the hypothesis that alpha band activity plays a role in gating attention through the inhibition of irrelevant information (for review see: Klimesch et al., 2007; Jensen and Mazaheri, 2010). Importantly, occipital alpha oscillations associated with visuospatial attention are thought to be under frontal and parietal control (Liu et al., 2016), and occipital alpha activity can be modulated by applying transcranial magnetic stimulation (TMS) to the frontal or parietal cortex (Capotosto et al., 2009; Sauseng et al., 2011; Marshall et al., 2015). This finding is in line with current theories that propose a crucial role for a frontoparietal network in the control of attention (Corbetta and Shulman, 2002; Noudoost et al., 2010).

One question that remains to be elucidated is how the frontoparietal attention network may control occipital alpha oscillations in the service of visuospatial attention. Recent evidence suggests that communication between distant brain regions might be facilitated through neuronal coherence, such that two regions that oscillate in-phase show increased communication, while two regions that oscillate out-of-phase are not able to communicate effectively (Womelsdorf and Fries, 2007; Sauseng and Klimesch, 2008). Importantly, there is evidence to suggest that the frontoparietal attention network might communicate through coherence in the alpha band. During visual attention tasks, previous studies have shown changes in alpha coherence as a function of attention between frontal and parietal cortex (Sauseng et al., 2005), between frontal and parietal-occipital cortex (Zanto et al., 2010; Doesburg et al., 2016), as well as between parietal and occipital cortex (Doesburg et al., 2009). Indeed, resting state data has shown that blood oxygen level dependent (BOLD) activity in the frontoparietal network correlates with alpha power (Sadaghiani et al., 2012). In addition, perturbing neural activity in a portion of the frontoparietal network, the inferior frontal junction, results in reduced attentional modulation of alpha band phase coherence between frontal and parietal-occipital cortex (Zanto et al., 2011). Despite research indicating a significant role of frontoposterior alpha coherence in visual attention, direct evidence that the frontoparietal network uses alpha coherence to bias visuospatial attention is lacking.

In the current study we aimed to test the effect of coherent frontoparietal alpha (8–12 Hz) stimulation on spatial attention. We adopted a transcranial alternating current stimulation (tACS) protocol (Polanía et al., 2012) that has been shown to increase coherence between two brain regions by stimulating these regions in phase (Helfrich et al., 2014a). Here we stimulated both the frontal and parietal cortex within the alpha band while participants performed a spatial attention task to assess whether this would subsequently increase phase coherence between these regions. Because tACS was applied to one hemisphere, we hypothesized that the stimulation would cause hemispheric-specific effects on top-down attention mechanisms. Specifically, we anticipated that in-phase alpha stimulation of the right frontal and parietal cortex would increase alpha band coherence in the right hemisphere. Moreover, previous studies found increased alpha coherence contralateral to the side of attention (Sauseng et al., 2005; Doesburg et al., 2009). Accordingly, we expected that the anticipated increase in coherence in the *right* hemisphere would be associated with improved attention to the *left* hemifield. We also investigated the effect of stimulation on anticipatory alpha power in parietal-occipital cortex. Previous research showed opposite effects of attention on alpha coherence and anticipatory alpha power (Sauseng et al., 2005; Doesburg et al., 2009). Based on these findings, the anticipated *increase* in right-hemispheric coherence might be associated with a *decrease* in anticipatory alpha power in the right occipital cortex.

## Materials and Methods

### Participants

Forty healthy, right-handed, participants with normal or corrected-to-normal vision were recruited for this experiment. Specifically, participants were selected based on the following criteria: between 18–35 years old; no neurological or psychiatric disorders; no substance abuse; not taking anti-depressants or anti-anxiety medication; no history of seizures; not pregnant; no color blindness; no glaucoma; no macular degeneration; no amblyopia; no strabismus; no metal anywhere in the head, excluding the mouth; no cardiac pacemakers; no implanted medication pumps; no electrodes inside the heart; no heart disease; no increased intracranial pressure; no family history of epilepsy; no epileptogenic medications; no hearing impairments. Two participants were excluded because of technical problems. One participant was excluded because she indicated that she ignored the attention cue during the experiment. Participants were randomly assigned to either the stimulation group (final sample: *n* = 18; 12 female; mean age = 24.8, SD = 3.2) or the sham group (final sample: *n* = 19; 10 female; mean age = 25.6, SD = 3.9). This study was carried out in accordance with the recommendations of the Institutional Review Board (IRB) of the University of California, San Francisco. All subjects gave written informed consent in accordance with the Declaration of Helsinki.

### Spatial Attention Task

**Figure 1:**
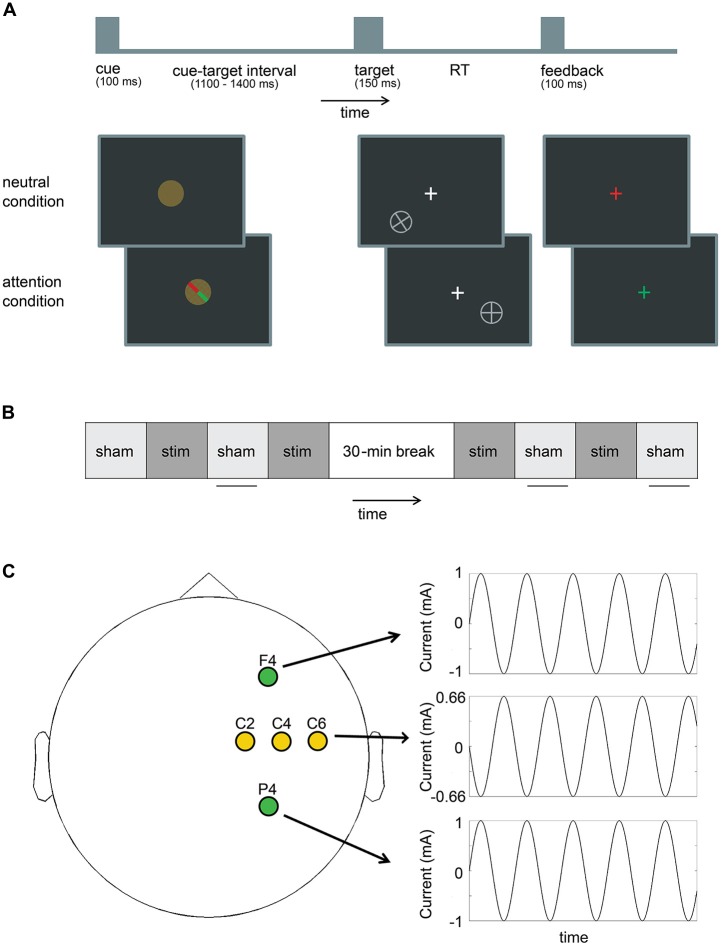
**(A)** Schematic overview of the spatial attention paradigm (not to scale). On each trial, participants were presented with a cue, and after a variable interval, with a target. In the neutral condition, the cue contained no information about the location of the target, while in the attention condition, the cue indicated whether the target would appear in the lower left or lower right quadrant of the screen on one of two fixed locations. Participants were instructed to indicate with a button response if the target was a plus sign, or a rotated plus sign. After a response was made feedback was presented and the next cue was presented 1500 ms after the onset of the last target. **(B)** The experimental design consisted of alternating blocks of sham (sham) and real stimulation (stim) with a 30-min break halfway through the experiment. (The sham group received sham stimulation on every block.) Horizontal lines indicate which blocks were included for the behavioral and electroencephalography (EEG) analysis. **(C)** F4 and P4 were stimulated in-phase at each person’s individual alpha frequency (IAF). C2, C4 and C6 were used as return electrodes and oscillated out-of-phase with F4 and P4.

The spatial attention task was presented via the Presentation software package (version 18; Neurobehavioral Systems, Inc.). Participants were seated in a dark room 65 cm from a Viewsonic G220FB monitor with illuminance of 95 cd/m^2^. Participants performed eight blocks of a cued spatial attention task. At the beginning of each trial, participants were randomly presented with either a 100% valid attention cue (100 ms) that indicated whether the upcoming target would appear left or right of the fixation cross, or a neutral cue that contained no spatial information about the upcoming target (Figure 1A). Differences in neural activity and behavioral performance between the attention and neutral cue serve to assess spatial selective attention processes. After a variable delay (1100–1400 ms), a target (150 ms) was presented in the lower left or lower right visual field, −135° or −45° relative to the central fixation cross, respectively, at a distance subtending 17° of the participant’s visual field of view from the central fixation cross. The fixation cross subtended 1° of the visual field of view, whereas the cue and target stimuli subtended 2°. The target was either a plus sign, which required a response with the right middle finger, or a rotated plus sign, which required a response with the right index finger. The magnitude of rotation was individually determined according to a staircase procedure in a separate thresholding block preceding the experiment. During the thresholding block participants performed 50 trials of the task during which the target rotation changed adaptively based on the participants performance. Target rotation (starting at 45°) decreased after every correct response and increased after an incorrect response. This staircase procedure was done to ensure accuracy was similar in the two groups at the start of the experiment (around 84% correct). Participants in the sham group were thresholded to an average rotation of 16.1°(±10.7°, range 3.1°–44.9°), while participants in the stimulation group were thresholded to an average rotation of 10.9°(±6.5°, range 1.9°–44.9°; No significant difference between the groups, *p* = 0.085.) Participants were instructed to keep their eyes on the fixation cross for the duration of the experiment and to covertly attend to the targets. Also, participants were instructed to respond to the targets as fast and accurately as possible. After a response was made, or if participants failed to respond within 1000 ms, feedback was presented to the participant. Feedback was positive (fixation cross turned green) if participants made the correct response within the response deadline of 1000 ms. Otherwise negative feedback (fixation cross turned red) was presented. The next trial/cue was presented 1500 ms after presentation of the target stimulus.

Trial parameters were pseudo randomized such that: (1) half of the trials were of the attention condition and half of the trials were of the neutral condition, and trials could not be of the same condition for more than four trials in a row; and (2) half of the trials showed the plus target and half of the trials showed the rotated plus target, and the same target could not be presented on more than four trials in a row. These parameters were randomized over the whole experiment (800 trials) without taking into account our blocked design. The side of target presentation was randomly chosen on each trial. *Post hoc* analysis showed that the average number of trials per condition per block was 12.5 (SD = 3) as predicted (100 trials per block/8 conditions). Importantly these numbers were the same for the stimulation and sham groups.

### Procedure

Before the start of the experiment, participants performed a short practice block (30 trials, ~1.5 min), followed by a thresholding block (50 trials, ~2.5 min). During the thresholding block we recorded electroencephalography (EEG) data to assess each participant’s individual alpha peak frequency (IAF). To this end, power spectra were calculated over a 2 s time window (1 s pre-target to 1 s post-target), and averaged over nine parietal and occipital electrodes (P7/P8/PO7/PO8/O1/O2/PO3/PO4/Oz). The IAF was determined as the peak frequency between 8 Hz and 13 Hz. In about half of the participants there was no clear peak in this frequency range (sham group: *n* = 8, stimulation group: *n* = 9), for those participants we used 10 Hz as the IAF (sham group: average IAF = 10.3 Hz, SD = 0.65 Hz, range = 9–12 Hz, stimulation group: average IAF = 10.3 Hz, SD = 0.67 Hz, range = 9–12 Hz). After thresholding, participants started the actual experiment, which consisted of eight blocks (100 trials, ~5 min/per block), with a 30-min break after four blocks (Figure 1B).

### Behavioral Analysis

Data were pooled over targets (plus sign or rotated plus sign), leaving four different conditions for analysis: (1) neutral cue/target in left hemifield; (2) neutral cue/target in right hemifield; (3) left attention cue/target in left hemifield; (4) right attention cue/target in right hemifield. For each subject and condition, we calculated accuracy (number of correct trials divided by the total number of trials; correct trials, incorrect trials and misses) and calculated the mean response times (RTs) from correct trials. Next, accuracy and mean RTs were analyzed using repeated-measures ANOVA (SPSS) with the within-participant factors of condition (attention cue vs. neutral cue) and side (target in left hemifield vs. target in right hemifield) and the between-participant factor of treatment (sham vs. stimulation). For consistency with the EEG analysis, only blocks during which sham was delivered were included in the analysis (see below). However, results were the same when all blocks were included. Table 2 shows the number of trials that were included for the behavioral analysis. Note that there was no significant difference in the number of trials included between the sham and stimulation group.

In addition, we performed an exploratory analysis to investigate the potential difference between online and offline tACS effects within the stimulation group. Specifically, we submitted mean reaction times from all eight blocks to an ANOVA with the within-participant factors stimulation (online vs. offline), condition (attention cue vs. neutral cue) and side (target in left hemifield vs. target in right hemifield).

### tACS

Stimulation was applied through a Starstim device (Neuroelectrics) using Ag/AgCl electrodes (Pistim) with a surface area of 3.14 cm^2^ each. The Common Mode Sense (CMS) and Driven Right Leg (DRL) electrodes of the Starstim device were attached to the right mastoid. Electrode impedances were held <10 kΩ. Our stimulation protocol was aimed at increasing alpha coherence between right frontal and parietal cortex. The right hemisphere was chosen because of the suggested right hemispheric dominance in attention (Heilman and Van Den Abell, 1980) and in particular, covert spatial attention (Wang et al., 2016). We placed our frontal stimulation electrode at F4 based on our previous findings that alpha phase coherence between electrodes in this region and visual cortex was increased during attention conditions (Zanto et al., 2010, 2011). Electrode F4 is roughly centered over the dorsolateral prefrontal cortex, which has been linked to top-down control of attention in the domain of feature-based attention (Zanto et al., 2011; Heinen et al., 2014), object-based attention (Clapp et al., 2010) and spatial attention (Giesbrecht et al., 2003; Sylvester et al., 2008). Additionally, we placed our parietal stimulation electrode at P4, which is roughly centered over the posterior parietal cortex. The posterior parietal cortex is known to be involved in both feature and spatial selective attention processes (Giesbrecht et al., 2003; Gilbert and Li, 2013). The electrodes at F4 and P4 were stimulated *in-phase* with a sinusoidal alternating current with 1000 μA peak amplitude (2000 μA peak-to-peak/current density of 0.32 mA/cm^2^) at each person’s IAF (Figure 1C). Return electrodes were placed at C2, C4 and C6 to spread the out-of-phase current over a larger area of non-interest. Each of these return electrodes received 666 μA current (1332 μA peak-to-peak), with an 180° phase offset relative to our electrodes of interest.

On each block, current was ramped up to the maximum strength over 15 s. For sham stimulation blocks, current was immediately ramped down again over 15 s, while for real stimulation blocks the current was maintained for the duration of the whole block (~5 min) before current was ramped down.

The sham group only received sham stimulation, while the stimulation group received alternating blocks of real stimulation and sham stimulation. Specifically, real stimulation was delivered during the second, fourth, fifth and seventh blocks (Figure 1B). This allowed us to record EEG data without tACS artifacts during the remaining blocks. Because there was no difference between the groups in terms of stimulation applied until the second block (i.e., both groups received sham during the first block), behavioral and EEG data analyses focused on blocks 2–8. To ensure group differences were not already present at the start of the experiment, control analyses were repeated with data from block one. Note that for this analysis, EEG data from one additional participant from the sham group was excluded because only one trial was left in the first block after the automatic trial rejection procedure (see below).

At the end of the experiment, we asked participants to report side effects on a scale from 1 (= not present) to 10 (= extremely noticeable). Importantly, there were no differences between the sham and stimulation groups in terms of side effect scores (Table 1). We also asked participants if they could tell any differences between the different blocks in terms of the stimulation. Eight participants from each group indicated that they experienced differences between blocks, which was not significantly different between groups (*χ*^2^ = 0.021, *p* = 0.886). Two participants (one in the sham group and one in the stimulation group) mentioned after the experiment that they periodically saw slight “flickering”, which could be due to partial stimulation of the optic nerve or retina. Critically, our main findings (e.g., between-group interactions) did not change after including/excluding these two participants.

**Table 1:** **Average scores on side effects on a scale from 1 (= not present) to 10 (= extremely noticeable)**.

Measure	Sham	Stimulation	*p*-value
Headache	1.7 ± 1.3	1.3 ± 1.0	0.369
Neck pain	1.2 ± 0.5	1.1 ± 0.3	0.781
Scalp pain	2.4 ± 1.7	2.6 ± 2.3	0.805
Tingling	5.1 ± 3.2	5.9 ± 2.7	0.398
Itching	4.3 ± 2.7	3.1 ± 3.1	0.219
Burning sensation	3.2 ± 2.6	3.1 ± 3.1	0.873
Skin redness	1.0 ± 0.0	1.0 ± 0.0	*
Sleepiness	3.6 ± 2.4	4.3 ± 2.9	0.429
Trouble concentrating	3.8 ± 2.9	2.8 ± 1.7	0.200
Acute mood change	1.1 ± 0.5	1.5 ± 1.2	0.189

Mean ± standard deviations are reported. *T-value cannot be computed because the standard deviations of both groups are 0.

### EEG Data Acquisition and Analysis

Electrophysiological signals were recorded at 500 Hz with Drytrodes (dry electrodes) using the wireless ENOBIO-20 system (Neuroelectrics) with no online filters. Data were recorded with CMS and DRL attached to either side of the right earlobe. Of the 20 electrodes, one electrode was used as an EOG electrode to record lateral eye movements (placed on the outer canthus of the left eye). The remaining 19 electrodes were distributed over the scalp with a relatively dense distribution over posterior cortex because of our interest in posterior alpha (FP1, FPz, FP2, F7, Fz, F8, T7, Cz, T8, P7, PO7, PO3, O1, Pz, Oz, O2, PO4, PO8, P8). Raw EEG data were analyzed using Fieldtrip^1^ (Oostenveld et al., 2011). Only data from the sham blocks were analyzed, matched across groups, to prevent any potential confound introduced by tACS artifacts. Data were epoched from 500 ms pre-cue to 1000 ms post-target and were demeaned/detrended and re-referenced to a common average reference. An independent component analysis was performed to remove components related to eye blinks. Trials containing artifacts or lateral eye movements were rejected using an automated procedure. Specifically, trials were excluded if one of the following conditions was met during the 1 s pre-target on one of our six electrodes of interest (see below) or the EOG channel: (1) the variance exceeded two times the standard deviation of the mean variance across trials; (2) the voltage exceeded 50 μV. The EOG channel was included to remove trials on which participants did not fixate properly in the pre-target period. For the sham group on average 20.1% of the trials were rejected (SD 6.7%), for the stimulation group on average 20.0% of the trials were rejected (SD 8.9%). The remaining trials were included for time-frequency analysis. (Note that these include trials on which participant made an incorrect response or no response. Data analysis focuses on the cue-target interval and we assume that participants use the cue to focus their attention on each trial according to the instructions).

Data from the cue-target interval were time-frequency analyzed using a fixed 200 ms sliding time window moving in steps of 50 ms. The data in each time window was multiplied with a Hanning taper and Fourier Transformed to give the spectral power at each latency. Power values were averaged over a time window from −400 to −100 ms pretarget and across a frequency range of IAF ± 2 Hz (width of frequency bins: 2 Hz). This time window was chosen because effects of attention need time to build up and are usually strongest towards the end of the cue-target interval. In addition, trimming the final 100 ms pre-target avoids contamination of the data by target-evoked responses. Data were averaged over trials for each condition separately (four conditions in total, see behavioral analysis) and electrodes, separately for three left parietal-occipital electrodes of interest (PO7/PO3/O1) and three right parietal-occipital electrodes of interest (PO8/PO4/O2). Averaged data were then submitted to a repeated-measures ANOVA with the within-participants factors of condition (attention cue vs. neutral cue), side (target in left hemifield vs. target in right hemifield) and hemisphere (left vs. right) and the between-participant factor of treatment (sham vs. stimulation).

### EEG Phase Coherence Analysis

Individual trial data from our time-frequency analysis were used for a coherence analysis. Phase-locking values (PLVs) were calculated for each condition separately using Fieldtrip and averaged over frequencies (IAF ± 2 Hz) and time (−400 ms to −100 ms target-locked; Lachaux et al., 1999). Next, we investigated hemispheric-specific effects of stimulation on frontoposterior connectivity. Our frontal stimulation electrode was positioned over F4. We were not able to record data from that electrode, so instead we assessed connectivity between F8 and our left and right parietal-occipital electrodes of interest. PLVs were averaged for the clusters of left (F8-PO7/F8-PO3/F8-O1) and right (F8-PO8/F8-PO4/F8-O2) electrodes separately. Data were submitted to a repeated-measures ANOVA with the within-participants factors of condition (attention cue vs. neutral cue), side (target in left hemifield vs. target in right hemifield) and hemisphere (left vs. right) and the between-participant factor of treatment (sham vs. stimulation).

## Results

### Behavioral Performance

Accuracy and RTs (Table 2) were each submitted to an ANOVA (see “Materials and Methods” Section). No main effects or interactions were found for accuracy data. Importantly, there was no difference between groups in terms of overall accuracy during the experiment (main effect of treatment *F*_(35)_ = 0.70, *p* = 0.409, $\eta_{\text{p}}^{2}$ = 0.02), indicating that our thresholding procedure worked well.

**Table 2:** **Summary of behavioral data within each group (mean ± standard deviation)**.

Measure	Response Time (RT) (ms)				Accuracy			
Cue	Neutral		Attention		Neutral		Attention	
Target	Left	Right	Left	Right	Left	Right	Left	Right
Sham	599 ± 85	590 ± 85	581 ± 85	571 ± 81	0.90 ± 0.09	0.89 ± 0.09	0.88 ± 0.10	0.90 ± 0.09
Stimulation	589 ± 55	595 ± 52	565 ± 55	577 ± 50	0.86 ± 0.13	0.86 ± 0.12	0.86 ± 0.13	0.86 ± 0.14

	**Number of trials included in RT analysis**				**Number of trials included in EEG analysis**			
	**Neutral**		**Attention**		**Neutral**		**Attention**	
	**Left**	**Right**	**Left**	**Right**	**Left**	**Right**	**Left**	**Right**

	65 ± 9	65 ± 9	70 ± 11	67 ± 9	57 ± 9	63 ± 9	60 ± 7	59 ± 6
	66 ± 13	66 ± 13	64 ± 11	64 ± 10	61 ± 10	60 ± 9	60 ± 11	59 ± 10

**Figure 2:**
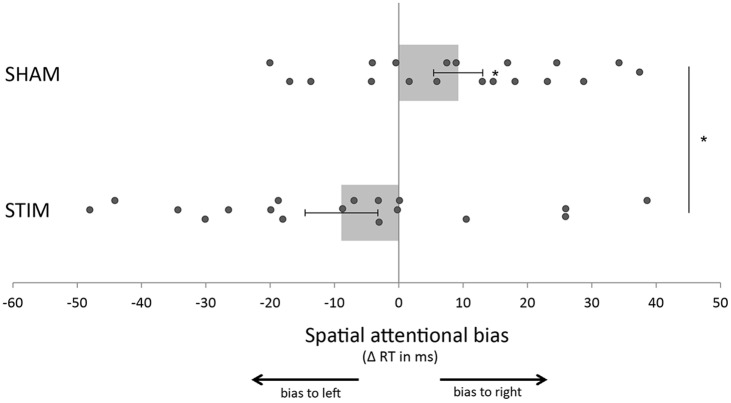
**Transcranial alternating current stimulation (tACS) shifted the spatial attention bias to the left compared to sham stimulation.** The spatial attention bias was calculated by subtracting the average response time (RT) for targets in the right visual field from the average RT for targets in the left visual field. Participants in the sham group (SHAM) showed a significant bias to the right (i.e., faster responses to targets in the right visual field compared to the left visual field) while no significant bias was present in the group that received real stimulation (STIM). Error bars represent standard error of the mean. Stars indicate a significant effect.

In terms of RTs we found a main effect of condition on RTs (*F*_(35)_ = 53.8, *p* < 0.001, $\eta_{\text{p}}^{2}$ = 0.61) such that participants responded significantly faster to targets that were preceded by a valid attention cue compared to targets that were preceded by a neutral cue. In addition, we found a side × treatment interaction (*F*_(35)_ = 7.2, *p* = 0.011, $\eta_{\text{p}}^{2}$ = 0.17), suggesting that our stimulation protocol significantly changed the spatial attention bias (Figure 2). Importantly, this interaction was not present in data from the first block (*F*_(35)_ = 0.3, *p* = 0.601, $\eta_{\text{p}}^{2}$ = 0.008), when both groups had only received sham stimulation.

To further investigate the side × treatment interaction found in post-stimulation blocks, we submitted data from each treatment group separately to a repeated measures ANOVA. These analyses showed that participants in the sham group were significantly faster on trials in which the target appeared in the right hemifield compared to trials on which the target appeared in the left hemifield (main effect of side *F*_(18)_ = 5.8, *p* = 0.027, $\eta_{\text{p}}^{2}$ = 0.25). Thus, the sham group exhibited a spatial attention bias towards target stimuli in the right hemifield (Figure 2). This was true regardless of whether the target was preceded by an attention cue or a neutral cue (condition × side *F*_(18)_ = 0.005, *p* = 0.946, $\eta_{\text{p}}^{2}$ < 0.001). In the stimulation group, such a spatial bias was not present (main effect of side *F*_(17)_ = 2.5, *p* = 0.133, $\eta_{\text{p}}^{2}$ = 0.13; Figure 2). Thus, the rightward spatial attention bias that was observed in the sham group, was not observed in the stimulation group. Of note, both groups showed a main effect of condition, in line with the results from the overall ANOVA (sham: *F*_(18)_ = 33.1, *p* < 0.001, $\eta_{\text{p}}^{2}$ = 0.65, stimulation: *F*_(17)_ = 23.2, *p* < 0.001, $\eta_{\text{p}}^{2}$ = 0.58). No other main effects or interactions were observed in the overall ANOVA or within group ANOVAs. In summary, compared to the sham group, stimulation altered a spatial attention bias.

Exploratory analyses were performed to assess whether stimulation effects differed between online blocks (during which tACS was applied) and offline blocks (during which sham was applied). Within the stimulation group, we found no stimulation × side interaction, thus the spatial bias did not differ between the blocks. This suggests that effects of tACS stimulation carried over to the following (sham) blocks. We did find a main effect of condition (*F*_(17)_ = 14.7, *p* = 0.001, $\eta_{\text{p}}^{2}$ = 0.46), as well as a stimulation × condition interaction (*F*_(17)_ = 8.2, *p* = 0.011, $\eta_{\text{p}}^{2}$ = 0.33). The latter was caused by the fact that the effect of condition was smaller in the stimulation blocks compared to the sham blocks. (Note that this effect was also present in the sham group when artificially dividing the blocks into sham and stimulation blocks matched with the blocks from the stimulation group. Thus, it seems like this stimulation × condition interaction is not caused by the stimulation, but is rather a result caused by subjects willing to focus their attention on some blocks more than others).

### Parietal-Occipital Alpha Power

Based on our* a priori* hypothesis, we submitted anticipatory alpha power from left and right parietal-occipital electrodes to an ANOVA (see “Materials and Methods” Section). This analysis showed a main effect of hemisphere (*F*_(35)_ = 4.5, *p* = 0.041, $\eta_{\text{p}}^{2}$ = 0.114), such that alpha power was higher in the right hemisphere compared to the left hemisphere. Even though the hemisphere × treatment interaction was only trending toward significance (*F*_(35)_ = 3.9, *p* = 0.058, $\eta_{\text{p}}^{2}$ = 0.099), we found that the main effect of hemisphere across groups was driven by the stimulation group. Specifically, *post hoc* analyses showed that the stimulation group showed a main effect of hemisphere (*F*_(17)_ = 4.7, *p* = 0.044, $\eta_{\text{p}}^{2}$ = 0.217), while no such effect was found in the sham group (*F*_(18)_ = 0.04, *p* = 0.85, $\eta_{\text{p}}^{2}$ = 0.002). In data from the first block, we found no main effect of hemisphere, or hemisphere × treatment interaction.

### Alpha Phase Coherence

**Figure 3:**
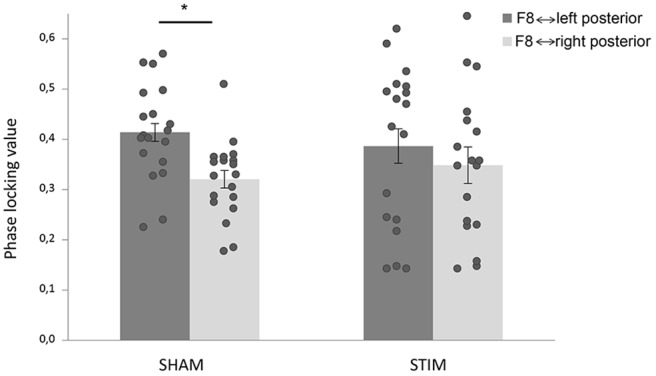
**tACS changed connectivity patterns in the alpha range.** Phase-locking values (PLVs) were averaged over time (−400 ms to −100 ms target-locked), and frequencies (IAF ± 2 Hz). Stimulation shifted frontoposterior alpha coherence; while the sham group showed stronger coherence between F8 and left parietal-occipital electrodes compared to F8 and right parietal-occipital electrodes, there was no difference in the stimulation group. Error bars represent standard error of the mean. The star indicates a significant effect.

We tested connectivity between right-sided F8 and left and right parietal-occipital electrodes to assess hemispheric-specific effects of stimulation between frontal and parietal-occipital regions. Pre-target PLVs in the alpha range were submitted to an ANOVA (see “Materials and Methods” Section). We found a main effect of hemisphere, such that F8 showed stronger phase coupling with left parietal-occipital electrodes compared to right parietal-occipital electrodes (*F*_(35)_ = 28.0, *p* < 0.001, $\eta_{\text{p}}^{2}$ = 0.45). Interestingly, we also found a hemisphere × treatment interaction (*F*_(35)_ = 5.04, *p* = 0.031, $\eta_{\text{p}}^{2}$ = 0.13; Figure 3). When this analysis was repeated with data from the first block we did find a main effect of hemisphere (*F*_(35)_ = 5.78, *p* = 0.022, $\eta_{\text{p}}^{2}$ = 0.145), but crucially, no hemisphere × treatment interaction (*F*_(35)_ = 0.6, *p* = 0.447, $\eta_{\text{p}}^{2}$ = 0.017).

For the post-stimulation blocks, *post hoc* ANOVAs within each group showed a main effect of hemisphere in the sham group (*F*_(18)_ = 47.3, *p* < 0.001, $\eta_{\text{p}}^{2}$ = 0.72), which was absent in the stimulation group (*F*_(17)_ = 3.2, *p* = 0.090, $\eta_{\text{p}}^{2}$ = 0.16). This result is in line with the effects of stimulation on RTs such that compared to the sham group, the stimulation group showed differences in a spatial bias. Within the stimulation group, we found no significant correlation between the hemispheric bias in coherence and the spatial attentional bias in behavior.

The stimulation group also showed a main effect of side (*F*_(17)_ = 4.6, *p* = 0.047, $\eta_{\text{p}}^{2}$ = 0.21), such that connectivity was overall higher for left hemifield targets compared to right hemifield targets.

## Discussion

In this study we aimed to test the effect of coherent frontoparietal alpha (8–12 Hz) stimulation on spatial attention. To this end, we employed a novel tACS protocol that was adapted from previous research demonstrating that alternating current stimulation increased coherence between brain regions (Polanía et al., 2012; Helfrich et al., 2014a). Compared to sham, in-phase frontoparietal alpha tACS was associated with differences in performance and neural outcome measures. Behaviorally, we found a significant difference in a spatial attention bias between the sham and stimulation group. Connectivity analyses further showed group differences in a hemispheric bias in functional connectivity between an electrode over the right frontal cortex and parietal-occipital electrodes. Taken together we interpret these results as confirmation that our stimulation protocol, which was aimed at increasing long-range alpha coherence, promoted a shift in a spatial attention bias via the modulation of alpha coherence. These results provide preliminary evidence that long-range alpha coherence is one mechanism by which the frontoparietal network controls spatial attention.

Our behavioral results showed that the sham group exhibited a significant attention bias to the right hemifield (i.e., faster responses to targets in the right hemifield compared to the left hemifield), while this effect was absent in the group that received stimulation. Although we can only speculate about the reason for a rightward bias in the sham group, we would like to emphasize that there was a significant difference between the groups in terms of the attention bias. This suggests that over and above any biases that might exist, stimulation altered the attention bias. It might be noted that in (to date unpublished) data that we recently collected, we replicate the finding of a rightward bias using a very similar task. In that study, we asked participants to respond bimanually to the target suggesting that the rightward bias is not related to the response hand.

In accordance with the behavioral effects, neural processing was different in the group that received stimulation. Connectivity analyses showed effects of stimulation on frontoposterior alpha coherence, between a right lateral prefrontal electrode (F8) and parietal-occipital electrodes. A hemisphere × treatment interaction was found, which was driven by the fact that the sham group showed significantly stronger connectivity between F8 and left posterior electrodes than between F8 and right posterior electrodes, while no difference was found in the stimulation group. A direct comparison of right hemispheric connectivity strength between groups did not reach significance, which could be (partly) due to the fact that we assessed coherence between different electrodes than the ones that were used for stimulation. Also there were large individual differences in connectivity strength (Figure 3). Nonetheless, the sham group exhibited significant lateralization in frontoposterior alpha coherence, which was not present in the stimulation group.

As described in the “Introduction” Section, we hypothesized that our stimulation protocol would increase alpha coherence in the right hemisphere and that this would be associated with increased attention to the left hemifield. Our findings are partially consistent with these hypotheses. While we did not observe significant effects in direct between-group comparisons, we did find a shift in the correct direction. That is, we found a relative increase in right hemispheric coherence (relative to left hemispheric coherence) and a shift in attention towards the left hemifield. In terms of anticipatory alpha power, we found that alpha power was significantly higher in the right parietal-occipital cortex compared to the left parietal-occipital cortex across the groups. We found no significant interaction effects, but a hemisphere × treatment effect was trending towards significance (*p* = 0.058), so if anything, stimulation increased alpha power in the right hemisphere and/or decreased alpha power in the left hemisphere. In the light of the current theories emphasizing a role of alpha in inhibition (Klimesch et al., 2007; Jensen and Mazaheri, 2010), a shift of alpha power towards the right hemisphere seems incongruent with a behavioral attention shift towards the left hemifield. However, previous studies also found that exogenous enhancement of alpha power was associated with an increase in performance (Klimesch et al., 2003; Helfrich et al., 2014b). In contrast to previous studies (Worden et al., 2000; Sauseng et al., 2005), we didn’t find a hemisphere × side × condition interaction, indicative of alpha lateralization prior to the cue. This interaction effect might have been obscured by the main effect of hemisphere, or might have been reduced (non-significantly) by the stimulation.

Our results are in line with previous studies (Sauseng et al., 2005; Doesburg et al., 2009, 2016; Zanto et al., 2010, 2011; Sadaghiani et al., 2012) that suggested a role for long-range alpha in visual attention. Moreover, studies in monkeys suggested that while feedforward communication is associated with gamma band activity, feedback communication is associated with alpha (van Kerkoerle et al., 2014) or beta band activity (Buschman and Miller, 2007; Bastos et al., 2015). These results suggest that our stimulation protocol might have altered feedback, or top-down processes. Nonetheless, further research is needed to clarify the range of frequencies that are involved in long-range feedforward and feedback communication. Furthermore, it should be noted that our effects cannot be attributed to the deployment of selective attention processes because we found no interaction effects with the attention condition. This suggests that the observed effects were similar for both attention and neutral cues. Thus, rather than modulating top-down resources that are allocated on a trial-by-trial basis as a function of the cue, it seems that stimulation caused a global shift in attention that was continuously present during the experiment.

It has been proposed that tACS modulates cognitive function via a combination of neural entrainment and resonance, which results in the recruitment of neurons into a local oscillating network that in turn affects both local and network level computations (Battleday et al., 2014). Previous studies have applied tACS in the alpha frequency to parietal/occipital cortex. They showed that it is possible to boost alpha power with tACS (Zaehle et al., 2010), with after-effects up to 70 min (Kasten et al., 2016). However, the effects might be state-dependent (Neuling et al., 2013; Ruhnau et al., 2016) and results on associated behavioral changes have been mixed. Specifically, Helfrich et al. (2014b) showed improved target detection after alpha tACS, while other studies found no effects (Neuling et al., 2013), or impaired target detection (Brignani et al., 2013). A study that combined a spatial attention task with an endogenous cue and alpha tACS over parietal cortex (Hopfinger et al., 2016), found no effect of stimulation. However, in this study we attempted to increase alpha band coherence, rather than increasing alpha power locally.

Stimulation electrodes were placed over F4 and P4, roughly corresponding to the dorsolateral and posterior parietal cortex. These are key regions in the frontoparietal attention network (Corbetta, 1998), and are known to be a source of top-down suppression signals that help prevent processing irrelevant information (reviewed in Zanto and Rissman, 2015). Despite the fact that we used small stimulation electrodes (3.14 cm^2^), the spatial resolution of tACS is limited because the current will spread and individual differences in anatomy make it hard to pinpoint the exact anatomical location of stimulation. Return electrodes were placed over the right motor and sensory cortex. This region has therefore also received potentially effective stimulation. The retina, which is known to be very sensitive to electrical stimulation, might also be affected (Laakso and Hirata, 2013). However, we would like to emphasize that even if the motor cortex, sensory cortex or retina, have received an effective dose of stimulation, this is unlikely to explain our results. The effects of stimulation on behavior were dependent on the spatial location of the stimulus. It is unlikely that stimulation of the aforementioned areas would selectively affect stimuli in the left or right visual hemifield, but rather it would have affected processing of all stimuli equally. Future fMRI studies using the same stimulation protocol could shed more light on the exact regions that showed neural changes due to the stimulation. In addition, it could assess how other measures of functional connectivity are affected by the stimulation protocol.

The current setup of the study brings some limitations to the interpretation of the results. First, we did not stimulate participants with a control frequency. Hence, the observed effects might not be selective to stimulation in the alpha range. Second, our crucial comparisons are between sham and in-phase stimulation. A recent study, using a similar setup showed that in-phase stimulation increased coherence between stimulation sites (Helfrich et al., 2014a). We also found hemispheric-specific changes in coherence after stimulation between regions close to the sites of stimulation. However, we cannot be certain our effects are caused by increased coherence because we didn’t include a control condition with out of phase stimulation. Alternatively, the observed effects could be due to neuroplastic changes of frontal and/or parietal cortex. Third, we focused analysis on sham blocks to prevent potential confounds introduced by tACS artifacts in the EEG data. A disadvantage of this approach is that the analysis is not sensitive to tACS effects that wear off quickly, but rather looks at offline effects of tACS. Behaviorally, we found no difference in spatial attention bias between sham and stimulation blocks within the group that received tACS. Nevertheless, we might not have been able to pick up more subtle differences that could have been assessed with EEG. Finally, based on our* a priori* analysis, we focused statistical analysis on changes in the alpha band. However, effects of stimulation might also be observed in other frequency bands.

In conclusion, we used a novel tACS protocol that differentially affected spatial attention in groups that received sham or real stimulation, both in terms of a behavioral spatial attention bias as well as associated neural measures. The results provide preliminary evidence that long-range alpha coherence plays an important role in top-down guidance of spatial attention. Future studies need to address the frequency-specificity and anatomical-selectivity of these effects as well as whether these results are specific to in-phase stimulation.

## Author Contributions

MRvS, TPZ and AG designed the study and wrote the article; MRvS acquired the data and analyzed the data.

## Funding

This research was supported by a National Institutes of Health (NIH) Grant R01MH096861 to AG and TPZ and a H2020 Marie Curie Fellowship to MRvS.

## Conflict of Interest Statement

AG is a scientific advisor for Neuroelectrics, a company that produces a tACS device. The other authors declare that the research was conducted in the absence of any commercial or financial relationships that could be construed as a potential conflict of interest.

## Footnotes

^1^http://www.ru.nl/neuroimaging/fieldtrip
